# Modified keyhole technique for the treatment of parastomal hernia: A case series

**DOI:** 10.1016/j.ijscr.2020.04.082

**Published:** 2020-05-12

**Authors:** Ryohei Ando, Ryuichiro Sato, Masaya Oikawa, Tetsuya Kakita, Takaho Okada, Takashi Tsuchiya

**Affiliations:** a Department of Gastroenterological Surgery, Sendai City Medical Center Sendai Open Hospital, 5-22-1 Tsurugaya, Miyagino-ku, Sendai, 983-0824, Japan; b Department of Surgery, Tohoku University Graduate School of Medicine, 1-1, Seiryo-machi, Aoba-ku, Sendai, 980-8574, Japan

**Keywords:** ePEFE, expanded polytetrafluoroethylene, Parastomal hernia, Sugerbaker, Keyhole, Mesh repair, Intraperitoneal

## Abstract

**Introduction:**

Parastomal hernia is one of the common complications of permanent stoma, and its incidence was nearly 50%. Surgical management is challenging and associated with relatively high recurrence rate. Mesh repair was demonstrated to reduce recurrence compared to non-mesh repair, and modified Surgerbaker and keyhole technique are the most cited intraperitoneal mesh repairs. In the keyhole technique, recurrence often occurs by herniation through the central hole. We present four parastomal hernia cases successfully repaired by modified keyhole technique, in which a cylinder-shaped synthetic mesh was attached to the keyhole mesh to cover the angle between the keyhole and the bowel.

**Presentation of cases:**

There were 1 male and 3 females with mean BMI of 25.7 kg/m^2^. Mean operative time was 114 min. There were two end-colostomies, one loop-ileostomy and one ileal conduit cases. Postoperative complication was observed in two cases, which was cerebral infarction and paralytic ileus. There were neither infectious complications nor seroma formation, and mean postoperative hospital stay was 18 days. With mean follow-up time of 36 months (range 10–66), we experienced no recurrence.

**Conclusions:**

Having lower recurrence rate, the modified Sugerbaker technique is considered preferable over the keyhole technique, but the bowel going to the stoma needs to be lateralized enough to be covered by relatively large mesh, which is not always accomplished. In such instances, our modified keyhole technique would be a feasible alternative.

## Highlights

• Parastomal hernia is one of the common complications of permanent stoma.
• Surgical management is associated with relatively high recurrence rate.
• Modified Sugarbaker and keyhole techniques are the most cited intraperitoneal mesh repairs.
• Our modified keyhole technique overcame the weakness of the keyhole technique.

## 1 Introduction

Parastomal hernia is one of the frequent complications of permanent stoma. The risk of developing parastomal hernia varies depending on the stoma types, with end-colostomy being the highest incidence of 4–48%, followed by loop colostomy (0–30.8%) and end-ileostomy (1.8–28.3%) [1]. Patients’ factors include obesity, malnutrition, steroid use, advanced age, and increased intra-abdominal pressure [1,2]. Parastomal hernia can be managed conservatively, and the indications for surgical repair are bulging, pain, obstruction, incarceration, and difficulty in attaching appliance [[2], [3], [4]].

Parastomal hernia repair includes primary repair, relocation and mesh repair, all of which are associated with recurrence. Although there is no standard procedure, mesh repair was demonstrated to have lower recurrence rate compared to primary repair, and the position of the mesh could be fascial onlay, preperitoneal-retromuscular, or intraperitoneal [4,5]. Modified Sugarbaker and keyhole techniques are the most cited intraperitoneal mesh repairs. The modified Sugerbaker technique is a variation of the technique originally reported by Sugarbaker in 1980 [6], and both hernia orifice and elevated intestine are widely covered by one mesh. In the keyhole technique, the elevated bowel is passed through a central hole and the mesh is fixed to the abdominal wall [3].

In the keyhole technique, recurrence often occurs by herniation through the central hole [3]. Here, we present four parastomal hernia cases successfully repaired by modified keyhole technique, in which a cylinder-shaped mesh was attached to the keyhole mesh to prevent recurrence. This is a retrospective, consecutive case series at our community hospital between 2013 and 2018. Written informed consent for publication of the patients’ clinical details and clinical images was obtained from the patients, and this study was approved by the Ethics Committee of the institution (#2019-0033). The research work has been reported in line with the PROCESS criteria for a case series [7].

## 2 Presentation of cases

### 2.1 Case 1

**Fig. 1:**
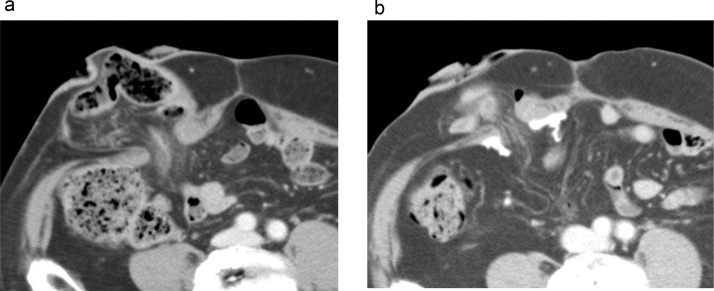
CT images of case 1. a. Preoperative abdominal CT showed herniation of the ileal loop into the subcutaneous space, compatible with parastomal hernia. b: CT image 2 years after the repair demonstrated no recurrence of parastomal hernia. There was no sign of mesh shrinkage or bowel obstruction.

Laparoscopic low anterior resection with diverting loop ileostomy was performed for a 77-year-old male rectal cancer patient. Severe anastomotic stricture which was refractory to endoscopic dilatation prevented ileostomy reversal. He developed parastomal hernia a year after the operation, which resulted in strangulated parastomal hernia necessitating emergency surgery several months later. Two years after the second operation, parastomal hernia repair was scheduled due to increasing bulging and pain around the stoma. Abdominal Computed tomography (CT) showed herniation of the ileal loop into the subcutaneous space, compatible with parastomal hernia (Fig. 1a).

**Fig. 2:**
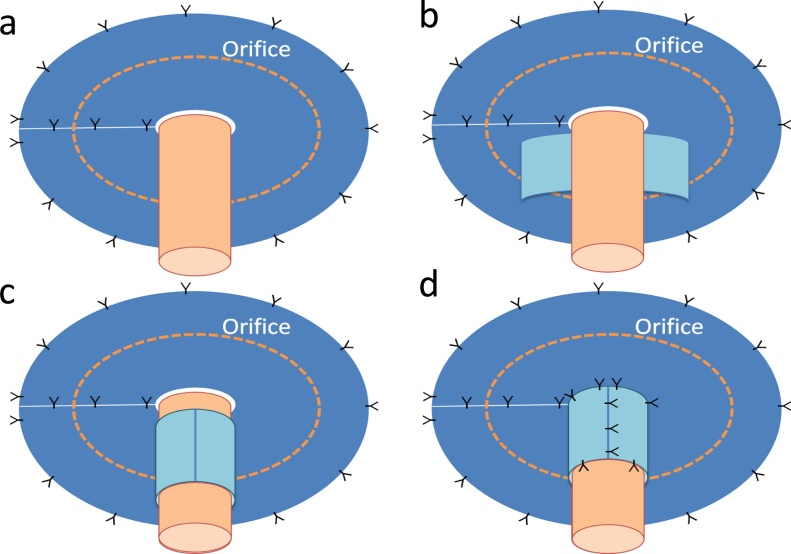
Modified keyhole technique. a: The hernia orifice was covered by elliptical ePTFE keyhole mesh while the elevated loop was passed through a cut-out hole. The mesh was fixed to the abdominal wall with interrupted 2-0 polypropylene monofilament sutures. b, c: The elevated intestine was wrapped using a rectangular ePTFE mesh. d: The wrapping mesh was secured to the keyhole mesh and to the elevated intestine with interrupted 2-0 polypropylene sutures.

**Fig. 3:**
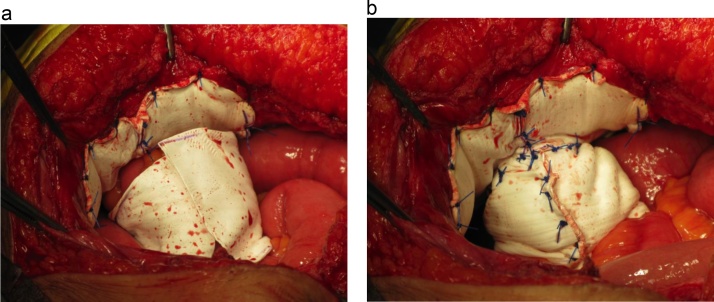
Intraoperative images of case 1. a: Using 15 × 10 cm elliptical expanded ePTFE mesh, the hernia orifice was covered while the elevated loop was passed through a cut-out hole. The mesh was fixed to the abdominal wall with interrupted 2-0 polypropylene monofilament sutures. Then the ileal loop was wrapped with 15 × 5 cm rectangular ePTFE mesh. b: The “cylinder” portion of the mesh was secured to the keyhole mesh and the bowel loop with interrupted 2-0 polypropylene sutures.

After skin preparation, a sterilized appliance was attached to the stoma to prevent contamination. Under general anesthesia, the abdomen was reopened with a midline incision and hernia contents were reduced. A large hernia orifice about 8 cm in size was noted around the loop ileostomy. Using 15 × 10 cm elliptical expanded polytetrafluoroethylene (ePTFE) mesh (Dualmesh; WL Gore & Associates, Newark, DE), the hernia orifice was covered while the elevated loop was passed through a cut-out hole. The mesh was fixed to the abdominal wall with interrupted 2-0 polypropylene monofilament sutures. Then the ileal loop was wrapped with 15 × 5 cm rectangular ePTFE mesh, and this “cylinder” portion was secured to the elliptical “keyhole” mesh with interrupted 2-0 polypropylene sutures. Finally, the cylinder and the bowel loop were fixed with interrupted 2-0 polypropylene (Fig. 2, Fig. 3).

Postoperative course was uneventful and the patient was discharged on the 7th postoperative day. The CT images 2 years after the repair demonstrated no recurrence of parastomal hernia, and there was no sign of mesh shrinkage or bowel obstruction (Fig. 1b). There was no apparent recurrence 3 years after the repair.

### 2.2 Case 2

**Fig. 4:**
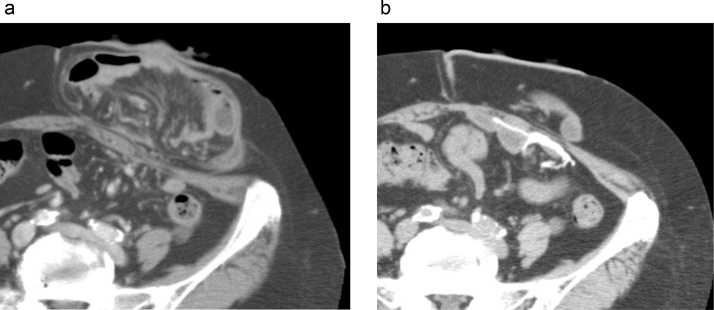
CT images of case 2. a. Preoperative abdominal CT demonstrated large parastomal hernia with herniation of the small intestine into the subcutaneous space. b: CT image 1 year after the repair demonstrated no recurrence of parastomal hernia.

A 76-year-old woman was referred to our hospital 14 years after total cystectomy and ileal conduit diversion performed for bladder cancer. She had suffered from bulging and pain around the ileal conduit, which aggravated over time. Abdominal CT findings were compatible with parastomal hernia (Fig. 4a).

**Fig. 5:**
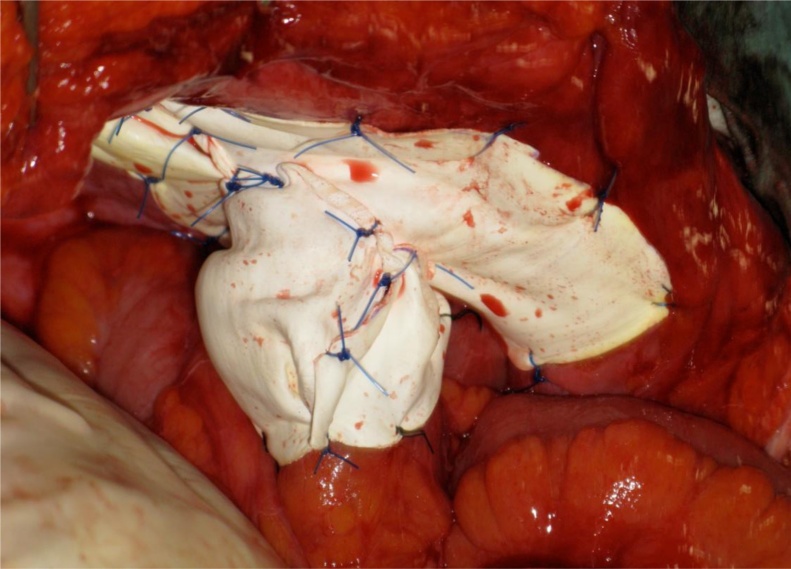
Intraoperative images of case 2. The hernia was repaired with modified keyhole technique using 15 × 10 cm elliptical and 12 × 4 cm rectangular ePTFE meshes.

Parastomal hernia repair was performed under general anesthesia. The abdomen was reopened with a midline incision and hernia contents were reduced. There was a large hernia orifice about 7 cm in size around the ileal conduit. The anastomosed ureter, as well as limited mobility of the conduit caused by adhesion, precluded performing the Sugarbaker procedure. The hernia was repaired with the modified keyhole technique using 15 × 10 cm elliptical and 12 × 4 cm rectangular ePTFE meshes (Fig. 5).

Postoperative recovery was complicated with paralytic ileus which resolved conservatively, and she was discharged on postoperative day 13. Abdominal CT confirmed no recurrence 1 year after the repair (Fig. 4b), and the patient has been free from hernia recurrence for 5 years.

We have performed the modified keyhole technique for 4 cases which were summarized in Table 1. Other than two cases described above, there were two end-colostomy cases. There were 1 male and 3 females with mean BMI of 25.7 kg/m^2^. Mean operative time was 114 min. A 75-year old women developed cerebral infarction on postoperative day 5. There were neither infectious complications nor seroma formation, and mean postoperative hospital stay was 18 days. With mean follow-up time of 36 months (range 10–66), we have experienced no recurrence.

**Table 1:** Four parastomal hernia cases repaired by modified keyhole technique.

No	age	sex	BMI (kg/m^2^)	original disease	stoma type	incisional hernia	ASA-PS	operative time (min)	bleeding (ml)	postoperative complication	postoperative hospital stay (days)	recurrence	observation time (months)
1	80	m	26.2	Rectal cancer	loop ileostomy	–	2	110	80	–	7	–	39
2	76	f	25.4	Bladder cancer	ileal conduit	–	2	127	170	ileus	13	–	29
3	75	f	28.9	Rectal cancer	end colostomy	–	3	111	100	cerebral infarction	42	–	66
4	64	f	22.3	Rectal NET	end colostomy	+	2	107	5	–	10	–	10

ASA-PS; American Society of Anesthesiologists physical status, BMI; Body Mass Index, NET: Neuroendocrine tumor.

## 3 Discussion

Parastomal hernia is one of the common complications of permanent stoma, and its incidence was reaching 50% for end-colostomy [1]. The majority of the hernia develops in the first year [5]. Surgical management is challenging and associated with relatively high recurrence rate. Mesh repair was demonstrated to reduce recurrence compared to non-mesh repair, but the result was still unsatisfactory [5,6]. Recently, prophylactic mesh reinforcement around the stoma site was demonstrated to reduce the incidence of parastomal hernia in randomized trials and meta-analysis [8,9]. Modified Surgerbaker technique and keyhole technique are the most cited intraperitoneal mesh repairs. The Sugerbaker technique had fewer recurrences than the keyhole technique, with reported recurrence rate of 5–15.4% and 27.9–72%, respectively [2,3,10]. Muysims et al. reported 8 recurrences out of 11 cases repaired with the keyhole technique, and the cause of recurrence was always herniation through the central hole [3].

Our modified keyhole technique was developed to reduce the recurrence rate of the keyhole technique. The construct covered the angle between the keyhole and the bowel, which theoretically overcome the weakness of the keyhole technique. Similar technique was reported by Fitzgerald et al. as “top hat” technique, since the construct resembled an inverted top hat. In their initial experience of 31 repairs, the recurrence rate was 19% with median follow-up of 31 months. Originally, the whole construct was constituted by xenograft mesh, then the technique was modified and synthetic mesh was used for the underlay portion of the construct after having experienced four recurrent cases. For fear of stoma obstruction, the “cylinder” portion was made from xenograft mesh which shrinks little. After the modification, the recurrence rate reduced to 13% with median follow-up of 22 months. They reported complication rate of 60% including one mesh infection and a case in which the xenograft mesh was resorbed, and resulted in recurrence [11]. Xenograft mesh was shown to have lower infection rate but usually costly [12]. In our series, we used solely ePTFE mesh since it is soft in texture, easy to manipulate, anti-adhesive, economical and readily available worldwide. The elliptical and rectangular mesh were cut-out from one large mesh. There has been a concern that ePTFE mesh might shrink which would give rise to enlargement of the keyhole resulting in recurrence [2,3], or narrowing of the keyhole resulting in cut-off intestine [10]. However, in the study of 815 laparoscopic incisional hernia repairs, the mean shrinkage rate of ePTFE mesh was 6.7% with median follow-up of 15 months [13]. Although the number of cases in our series was small, the mean observation time was 36 months which exceeded the time of highest recurrent risk. It is encouraging that we have not experienced recurrence, stoma obstruction or other mesh-related complications.

Having lower recurrence rate, the modified Sugerbaker technique is considered preferable over the keyhole technique, but the bowel going to the stoma needs to be lateralized enough to be covered by relatively large mesh. In cases with ileal conduit or loop ileostomy, or with dense adhesion, lateralization might not be accomplished. In such instances, our modified keyhole technique would be a feasible alternative. With the success of laparoscopic mesh repair for incisional hernia, reports of laparoscopic parastomal hernia repair have been increasing, and both modified Sugerbaker and keyhole techniques were shown to be performed safely [2,3]. Since our technique is a modification of the keyhole technique, it might be performed laparoscopically by experienced surgeons.

In summary, we presented our experience of parastomal hernia cases successfully managed with the modified keyhole technique. Although the technique seemed promising, studies with a larger cohort and longer follow-up are warranted.

## Declaration of Competing Interest

The authors declare that there are no conflicts of interest.

## Sources of funding

This report did not receive any specific funding from public, commercial, or not-for-profit sectors.

## Ethical approval

This study was approved by the Ethics Committee of the institution (#2019-0033).

## Consent

Written informed consent was obtained from the patients for publication of this case series and accompanying images. A copy of the written consent is available for review by the Editor-in-Chief of this journal on request.

## Author contribution

RA and RS performed the literature search and drafted the manuscript. RS MO, TK, TO, and TT participated in the critical revision of the manuscript. All the authors have read and approved the final manuscript.

## Registration of research studies

1. Name of the registry: UMIN-ICDR
2. Unique identifying number or registration ID: UMIN000040238.
3. Hyperlink to your specific registration (must be publicly accessible and will be checked): https://upload.umin.ac.jp/cgi-bin/ctr/ctr_view_reg.cgi?recptno=R000045907.

## Guarantor

Ryuichiro Sato and Takashi Tsuchiya.

## Provenance and peer review

Not commissioned, externally peer-reviewed.

## References

[1] Carne P.W., Robertson G.M., Frizelle F.A. (2003). Parastomal hernia. Br. J. Surg..

[2] DeAsis F.J., Lapin B., Gitelis M.E., Ujiki M.B. (2015). Current state of laparoscopic parastomal hernia repair: a meta-analysis. World J. Gastroenterol..

[3] Muysoms E.E., Hauters P.J., Van Nieuwenhove Y., Huten N., Claeys D.A. (2008). Laparoscopic repair of parastomal hernias: a multi-centre retrospective review and shift in technique. Acta Chir. Belg..

[4] Hansson B.M., Slater N.J., van der Velden A.S., Groenewoud H.M., Buyne O.R., de Hingh I.H. (2012). Surgical techniques for parastomal hernia repair: a systematic review of the literature. Ann. Surg..

[5] Al Shakarchi J., Williams J.G. (2014). Systematic review of open techniques for parastomal hernia repair. Tech. Coloproctol..

[6] Sugarbaker P.H. (1980). Prosthetic mesh repair of large hernias at the site of colonic stomas. Surg. Gynecol. Obstet..

[7] Agha R.A., Borrelli M.R., Farwana R., Koshy K., Fowler A., Orgill D.P., SCARE Group (2018). The PROCESS 2018 statement: updating consensus preferred reporting of CasE series in surgery (PROCESS) guidelines. Int. J. Surg..

[8] Brandsma H.T., Hansson B.M., Aufenacker T.J., van Geldere D., Lammeren F.M., Mahabier C. (2017). Prophylactic mesh placement during formation of an end-colostomy reduces the rate of parastomal hernia: short-term results of the dutch PREVENT-trial. Ann. Surg..

[9] Pianka F., Probst P., Keller A.V., Saure D., Grummich K., Büchler M.W. (2017). Prophylactic mesh placement for the PREvention of paraSTOmal hernias: the PRESTO systematic review and meta-analysis. PLoS One.

[10] Näsvall P., Rutegård J., Dahlberg M., Gunnarsson U., Strigård K. (2017). Parastomal hernia repair with intraperitoneal mesh. Surg. Res. Pract..

[11] Fitzgerald M.J., Ullrich S., Singh K., Misholy O., Kingham P., Brady M.S. (2018). Parastomal hernia repair using the “top hat” technique – an initial experience in 30 patients at Memorial Sloan Kettering Cancer Center. Am. J. Surg..

[12] Darehzereshki A., Goldfarb M., Zehetner J., Moazzez A., Lipham J.C., Mason R.J. (2014). Biologic versus nonbiologic mesh in ventral hernia repair: a systematic review and meta-analysis. World J. Surg..

[13] Carter P.R., LeBlanc K.A., Hausmann M.G., Whitaker J.M., Rhynes V.K., Kleinpeter K.P. (2012). Does expanded polytetrafluoroethylene mesh really shrink after laparoscopic ventral hernia repair?. Hernia.

